# Five Strategies Leaders in Academic Medicine Can Implement Now to Enhance Gender Equity

**DOI:** 10.2196/47933

**Published:** 2023-06-13

**Authors:** Jessica M Allan, Amber K Brooks, Cindy Crusto, Lauren D Feld, Amy S Oxentenko, Nancy D Spector, Monica Verduzco-Gutierrez, Julie K Silver

**Affiliations:** 1 Department of Pediatric Hospital Medicine Palo Alto Medical Foundation Palo Alto, CA United States; 2 Department of Pediatrics Stanford University School of Medicine Palo Alto, CA United States; 3 Department of Anesthesiology Wake Forest University School of Medicine Winston-Salem, NC United States; 4 Yale School of Medicine New Haven, CT United States; 5 Division of Gastroenterology & Hepatology UMass Chan Medical School Worcester, MA United States; 6 Division of Gastroenterology and Hepatology Department of Internal Medicine Mayo Clinic Rochester, MN United States; 7 Executive Leadership in Academic Medicine Department of Pediatrics Drexel University College of Medicine, Drexel University Philadelphia, PA United States; 8 Department of Rehabilitation Medicine University of Texas Health Science Center at San Antonio San Antonio, TX United States; 9 Department of Physical Medicine and Rehabilitation Harvard Medical School Boston, MA United States

**Keywords:** gender equity, diversity, leadership, academic medicine, gender, medicine, women in medicine, strategies, leadership, equity

## Abstract

Abundant disparities for women in medicine contribute to many women physicians considering leaving medicine. There is a strong financial and ethical case for leaders in academic medicine to focus on strategies to improve retention. This article focuses on five immediate actions that leaders can take to enhance gender equity and improve career satisfaction for all members of the workplace.

## Introduction

Disparities for women in medicine are abundant, and the pandemic had a profoundly negative effect, especially for women with minoritized intersection identities (eg, race and ethnic minority groups) [1]. Unfortunately, the high costs associated with faculty recruitment generally far exceed financial investments in retention. One academic institution reported the cost of physician recruitment and start-up was US $268,000-$957,000 per doctor based on specialty, experience, and expertise [2,3]. Work environments that are hostile to women physicians may also lead to them reducing their work hours, and this too has major financial ramifications for organizations. Despite the strong business case for investing in physician well-being and retention, many institutions have markedly underinvested in these areas. Not surprisingly, department chairs and other leaders are asking this critical question: “What can I do now to recruit and retain women faculty and demonstrate a stronger commitment to gender equity?”

In this article, we highlight five strategies that leaders in academic medicine can implement now to improve gender equity in the workplace. The eight authors of this paper represent a diverse group of women (eg, race/ethnicity, specialty, geography, age and career stage, and leadership roles) who have extensive experience and scholarship in diversity, equity, and inclusion (DEI). Our proposed list of strategies is not intended to be comprehensive as there are other excellent resources available [4,5], and it might not be applicable to all institutions depending on unique organizational factors for which we cannot account. However, with appropriate adaptation, we provide a basic framework to initiate crucial conversations and interventions. The proposed approaches should be strategically incorporated within a larger DEI plan—one that extends to all women and those from minoritized groups, and can be adapted to nonacademic settings.

## Conduct Stay Interviews

Stay interviews are often a missing component of faculty retention efforts; however, they have been used in corporate settings for many years to reduce employee turnover. In medicine, stay interviews have recently gained traction, particularly for the retention of nurses [6,7] and those underrepresented in medicine [8]. They are ideally conducted in person and involve one-on-one meetings with a supervisor. The supervisor follows a predetermined script that focuses on learning about the faculty member’s interests and goals, reinforcing the faculty member’s value to the organization, expressing with intentionality the desire for the individual to stay, inquiring about pain points (ie, stress/conflicts), and communicating the benefits of being a member of the department/division. Ideally, this is a free-flowing conversation (not a survey or other written format) that can occur during a formal process (eg, annual review) or informal setting (eg, over coffee). The supervisor guides the conversation but mostly listens (eg, 20% talking and 80% listening). Below is an example of a script:

- “Ideally, how would you like to see your career progress over the next couple of years?”
- “What talents and skills do you have now, or do you want to develop, that we can better support?”
- “What do you see as the biggest challenge you face when it comes to your work?”

All DEI efforts are at risk of being called “superficial” or “performative,” so instead of announcing to faculty “We are going to conduct Stay Interviews,” give them some context, such as:

You are all really valuable members of our department/division, and as part of our faculty retention efforts, we are going to conduct Stay Interviews [explain what these are]. Importantly, this is one of several new initiatives aimed at creating a supportive and equitable work environment. Our goal with Stay Interviews is to foster a better understanding of what is important to each of you so that we can support you now and in the future.

## Analyze Department/Division-Level Faculty Metrics

A commitment to gender equity and its strategic components must include the collection, analysis, and reporting of metrics; otherwise, the commitment is merely a token gesture. Metrics present an opportunity to identify gaps, understand what is working and what can be improved, and guide interventions and measure change (Textbox 1). There should be a defined timeline instituted for gathering and assessing metrics.

As the data are analyzed and gaps are revealed, we acknowledge that some implicit (unconscious) biases may be uncovered. To ensure that information is shared in a thoughtful and transparent manner and to avoid any potential for shaming individuals, it is important to approach this process with care for all involved (eg, deidentified aggregate data may be best in some instances). The ultimate goal is to inform decision-making and promote equity in a respectful and inclusive way. This information can be used during the faculty stay interviews when various topics come up to educate an individual faculty member about what the department/division is doing to address inequities as they relate to that individual. Moreover, the data can serve as a valuable resource for institutional leadership, providing insights that can inform change.

**Metrics to assess gender equity.**

Conduct analyses of the following as they relate to faculty (all metrics should include financial analysis, eg, honoraria and other financial payments or support for speakers):

- Pay
- Rank promotion (include an analysis of tenure, endowed professorships); data could also include feedback from decision makers on why faculty were deemed “not ready” [9]
- Nonrank promotion leadership (eg, directors, vice-chairs)
- Productivity (eg, clinical, research, intellectual property; include metrics such as relative value units and bibliometrics)
- Clinical, research, and administrative support (eg, dedicated human resources, space)
- Speaker invitations (include a subanalysis for the most prestigious lectures such as named lectureships, visiting professorships)
- Awards
- Philanthropic funding
- Discretionary financial support (eg, chair’s discretionary fund, sundry fund)
- Committee assignments and other citizenship tasks

These are examples and are not intended to be a complete list. They are focused on the department/division level in academic medicine. Consider not only gender analyses but other identity factors such as race/ethnicity. Additionally, consider subanalyses by terminal degree (eg, physicians including MD/PhDs vs PhDs). Analyses should be conducted on a regular basis.

## Implement Best Practices for Parental Leave and Return Policies

Parental leave policies should be readily available to all employees and presented at the time of the interview process. Sharing this information upfront alleviates the need for recruits to ask about parental leave, assists employees with family planning, and demonstrates a supportive culture for employees who want to have children [10]. Parental leave policies should be accessible, consistent, and applicable to everyone in the workplace, including birthing and nonbirthing parents, and those adopting or fostering [11]. The leave should be of adequate duration to support the physical and mental health benefits for parents and children. A minimum duration of 12 weeks of paid leave is recommended by the American Academy of Pediatrics and other medical societies [12]. More than 10 states currently mandate 12 weeks of paid family leave, and national legislation is being considered; therefore, it is prudent to ensure that departmental leave policies are legally compliant [13].

Policies need to be transparent regarding the compensation for leave and how time away from the practice is adjusted (eg, call, practice targets, relative value unit [RVU] expectations). Additionally, protecting time for prenatal and postnatal care is essential [14]. Depending on the specialty, reference to modifications that can be made (eg, exposure to fluoroscopy, hours of standing) should be noted [15].

Return-to-work policies following parental leave are essential to improve retention as physicians adjust to balancing career and childcare responsibilities. These policies can address the transition back into the workplace (eg, timing of call, schedule adjustments, flexible hours) and lactation needs. Flexible work policies are beneficial for balancing childcare responsibilities [16]. A lactation policy should ensure that adequate time is regularly blocked throughout the workday to ensure milk production is maintained, and time away should be accounted for by lactation RVUs so that the compensation is not negatively impacted by lactation needs [17]. Appropriate lactation spaces in proximity that offer both privacy and refrigeration services are essential.

## Invite Midcareer Women Faculty to Publish With Senior Faculty

Women faculty in academic medicine are less likely than men to be promoted to the rank of associate or full professor or to be appointed to leadership positions [18]. While women’s work-life choices may explain some of the differences in upward mobility, insufficient opportunities to engage in traditional scholarly activity that leads to promotion (eg, speaking invitations and publishing journal articles) remain significant barriers [19]. Women with with minoritized intersection identities (eg, race and ethnic minority groups) [20] (eg, women who identify with race, ethnic, sexual orientation, or gender minority groups) often carry the additional responsibility of developing and implementing DEI initiatives, mentoring trainees of color, and participating in community engagement activities [21]. While there are efforts at some institutions to revise the qualifications for promotion and advancement, most institutions still place a high value on publications, especially for midcareer women faculty (eg, more than 7 years as an assistant professor or rank of associate professor) [22].

As a strategy for supporting the advancement of midcareer women via the promotion process in academic medicine, it is imperative that women receive equitable opportunities to publish. We recommend conducting an analysis of publications by faculty over the past 5 years. How often were women (vs men) in their department/division invited to be middle authors or coauthors? Next, perform a secondary analysis to assess the first and last (senior) authorship status. Assessments should also specifically focus on gaps in authorship for midcareer women. This analysis may uncover some role models (eg, men who are intentional about collaborating with women in their department/division) as well as some implicit bias (eg, men who are not collaborating with women in their department/division), which is an opportunity to engage them in allyship. Consider developing a strategy to invite women to be middle authors, which allows them to share keen insights in a way that may be less onerous than the responsibility of the first or last author. If she does not have experience as a senior author, it is invaluable to receive an invitation from a senior colleague to share the last (senior) authorship. Mentorship through the entire process (ie, idea development, writing, editing, submission, and revision) will ensure her success [23].

## Avoid Bias in Faculty Evaluations

Many traditional indicators used to evaluate faculty work or competence in the appointments and promotions process show evidence of gender bias. For example, women, particularly those who are middle-aged, do not conform to gender stereotypes, and are in male-dominated departments, are more likely to receive lower teaching evaluations than men [24-26]. Studies have also demonstrated bias against women on other indicators of faculty success, including promotion, publications, pay [27], National Institutes of Health funding, honorifics [28], and reference letters for medical faculty hiring [29]. The degree to which teaching evaluations and other indicators are used and weighted as measures of competence in hiring, promotion, and tenure decisions could result in discriminatory practices. Leaders should acknowledge the existence and the systematic nature of gender bias [30]. With respect to teaching evaluations, leaders can take several actions to use learner ratings more responsibly: conceptualize evaluations as measures of student perceptions of instruction rather than a measure of teaching effectiveness or quality or course quality; use multiple methods to assess instruction; improve survey response rates because low response rates call into question the validity or truthfulness of the data; interpret the results, particularly qualitative comments, with caution; use evaluations to compare an individual’s teaching over time but not to compare to other faculty; and provide instructions on the survey that alert learners to potential bias in the rating process [26].

Participating in rigorously evaluated and effective interventions that break the gender bias habit [31] is an important step in eliminating bias in the evaluation process. Finally, consider the adaptation of the Holistic Review in Admissions Model [32] developed by the Association of American Medical Colleges [33]. This selection process can be applied to faculty by balancing academic metrics with experiences and attributes, and could address unique experiences that often disadvantage women, such as the “minority tax” and “citizenship burdens” [34-36].

## Conclusion

There is an urgent need to provide greater support for and investment in the academic medicine workforce. In this article, we highlighted five strategies that leaders can implement now to address gender equity for faculty members. These include conducting stay interviews, analyzing faculty metrics, supporting best practices for parental leave, inviting midcareer women to publish with senior faculty, and avoiding bias in faculty evaluations. Thoughtful and frequent communication with all key players is critical to inform, align, and support everyone. The strategies should be incorporated as part of more comprehensive and ongoing DEI efforts.

## Abbreviations

DEI: diversity, equity, and inclusion
RVU: relative value unit
